# “I’d like to let people know what we did:” values of Fukushima medical students following the Great East Japan Earthquake

**DOI:** 10.1186/s12909-022-03887-6

**Published:** 2023-04-15

**Authors:** Anna Stacy, Marcia Lange, Craig L. Katz, Satoshi Waguri, Robert Yanagisawa

**Affiliations:** 1 Department of Emergency Medicine, Icahn School of Medicine at Mount Sinai, 1 Gustave L. Levy Pl, New York, NY 10029 USA; 2 Department of Medical Education, Icahn School of Medicine at Mount Sinai, 1249 Park Avenue, New York, NY 10029 USA; 3 Departments of Psychiatry and System Design & Global Health, Icahn School of Medicine at Mount Sinai, 1 Gustave L. Levy Pl, New York, NY 10029 USA; 4 International Exchange Affairs, Fukushima Medical University, 1 Hikariga-Oka, , Fukushima-Shi, 960-1295 Japan; 5 Department of Internal Medicine, Icahn School of Medicine at Mount Sinai, 1 Gustave L. Levy Pl, New York, NY 10029 USA

**Keywords:** Medical school, Medical students, Natural disasters, Medical specialty, Radiation stigma, Fukushima Medical University

## Abstract

**Background:**

The Great East Japan Earthquake and the resulting tsunami and nuclear disaster on March 11, 2011 have had a profound and lasting effect on residents of Japan’s Fukushima Prefecture, particularly among evacuees. While there continues to be extensive news coverage and academic study of Fukushima Prefecture’s recovery, there has been little exploration of individual narratives. This study aims to illuminate some individual stories of medical students at Fukushima Medical University (FMU) who lived in the Prefecture at the time of the Earthquake.

**Methods:**

A qualitative approach was taken in order to investigate individuals’ experiences with the goal of adding a personal dimension to quantitative studies on the subject. 10 open-ended ethnographic interviews were conducted with medical students at FMU in years 1–5 who lived in Fukushima Prefecture at the time of the Great East Japan Earthquake. All interviews were audio recorded and transcribed. Transcriptions were reviewed using inductive thematic analysis under the lens of ethnographic anthropology.

**Results:**

Three major themes emerged from these interviews: first, that the events following the Earthquake influenced not only these students’ decisions to pursue careers in medicine, but the ways in which they hope to practice medicine in the future. Second, that these students were motivated to share their experiences by a want to change Fukushima Prefecture’s public image. And lastly, that the students viewed the opportunity to discuss their experiences through these interviews as healing, both for themselves and for the future.

**Conclusions:**

While multiple factors undoubtably contributed these students’ medical education, they cite the Earthquake as essential to their approach to their medical careers. Additionally, opportunities for the participants to discuss their experiences following the Earthquake appear to be rare but valued, as the students view their stories as their “legacies.” The enduring, burdening effects of the Earthquake appear to have galvanized the participating students to act on behalf of their communities and their Prefecture. Further qualitative studies in more generalizable populations are needed to improve and deepen our understanding of the societal, cultural, and personal impacts of the Great East Japan Earthquake.

## Background

There is no shortage of media coverage following disasters, and no shortage of criticisms of the media coverage following disasters. It is a familiar pattern: the first media wave reporting on the event; the second wave of clarifications and professional opinions; then the third, a stream of splashy, sensationalist pieces that play towards a hungry audience’s desire for more information. It all becomes so big and blurry.

This is my memory of the media coverage of the 2011 earthquake, tsunami, and nuclear plant explosion in Fukushima, Japan, and my only concept of Fukushima prior to visiting for research. News stories of contaminated fish, mutated plants, terrified and irradiated evacuees. There was no sense of personal narrative – people lost control of their own stories. The same is true in the academic literature; 8 years later, ethnographic studies of the Great East Japan Earthquake (GEJE) are extremely few.

During my time at Fukushima Medical University (FMU), my hope was to hear the experiences of students who were there when it happened. As half of my stay in Fukushima overlapped with the university’s exam period, I imagined that students would be reluctant to speak with me for fear of compromising their studying, and that the interviews I did manage to schedule would be very brief. However, the students were more than eager to share both their time and their stories. While I initially came to FMU to pursue questions of ethics in post-disaster research, I quickly found that the participants were more passionate about other topics related to the Great East Japan Earthquake, and I was excited for the discussions to shift. Therefore, a new research focus emerged and I simply began asking participants, “What was it like?” This inductive and discovery-based approach allowed me to reframe and refine the objectives for this study.

While there continues to be extensive news coverage and quantitative studies on Fuksuhima Prefecture’s recovery, there remains a paucity of scientific literature on individual narratives during and following the GEJE. A holistic understanding of such an impactful event requires investigations from both qualitative and quantitative perspectives. Through open-ended interviews, this study aims to illuminate individual stories of medical students at FMU who lived in the Prefecture at the time. Here, we utilize a qualitative approach based in ethnography to delve into their complex lived experiences, explore their responsive belief systems and coping strategies as a result of the Earthquake, and lastly provide a space in which survivors can control the narrative in academic research.

**Fig. 1:**
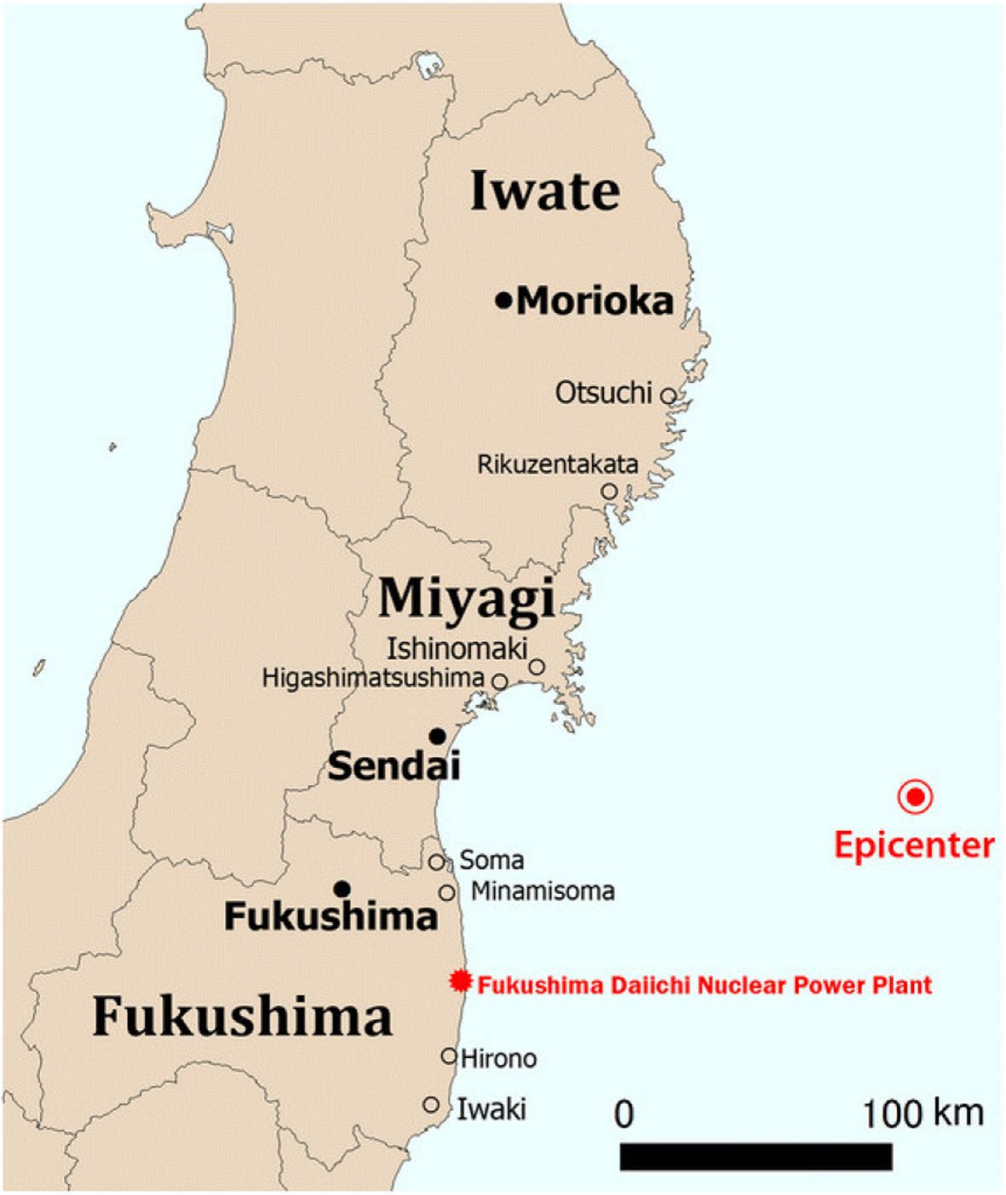
Locations of Iwate, Miyagi, and Fukushima Prefectures relative to the Daiichi Nuclear Power Plant and tsunami epicenter https://www.researchgate.net/figure/Three-severely-affected-prefectures-of-the-Tohoku-region-Japan-Iwate-Miyagi-and_fig2_281415756

In the afternoon of March 11^th^, 2011, a magnitude 9.0 earthquake off the coast of Japan’s Tōhoku region induced a major tsunami. This earthquake – the Great East Japan Earthquake – was the most powerful ever recorded in Japan, and the fourth most powerful recorded worldwide [1]. The resulting 6–8 m tsunami hit areas of Iwate Prefecture, Miyagi Prefecture, and Fukushima Prefecture (Fig. 1), causing enormous loss of life, as well as tremendous destruction of buildings, roads, and railways. As of December 10, 2019, the National Police Agency of Japan has declared 6,157 injured, 2,529 missing, and 15,899 dead [2]. As it reached the Fukushima Daiichi Nuclear Power Plant in Ōkuma, Fukushima Prefecture, the tsunami swept over the plant’s seawall, flooding the lower grounds and knocking out the emergency generators, which were required to cool the reactors. In the absence of cooling, 3 of the plant’s 4 units went into nuclear meltdown resulting in three hydrogen explosions which released radioactive material [3].

In the days following the accident, radiation released into the atmosphere lead the government to declare an evacuation zone around the plant, which grew to reach a 20-km radius [4]. An estimated 154,000 residents evacuated from the towns surrounding the plant to avoid contamination and relocated throughout Japan, principally in the central and western areas of Fukushima Prefecture [1]. There were significant challenges in evacuating patients from hospitals and nursing homes: the deaths of 51 “seriously ill patients” – e.g. terminally ill, suffering from dementia, requiring dialysis, and/or immobile – were attributed to insufficient transportation, inadequate medical facilities, lack of accompaniment by medical staff, improper heating, inappropriate seating for patients with limited mobility, and extremely prolonged travel due to the tsunami’s destruction of roads and bridges [5].

As of 27 February 2017, the Fukushima Prefecture government identified 2,129 “disaster-related deaths [6]” – this figure does not distinguish between the deaths of people displaced by the nuclear disaster compared to those displaced by the earthquake or tsunami, but media analysis suggests that 1,368 of these deaths are attributable to the nuclear evacuation [7]. While today, the evacuation zone is gradually shrinking and some residents are returning to their homes for the first time in 8 years, many still remain in temporary housing, or else have decided not to go back.

Despite ongoing fear regarding the potential health effects of the disaster, studies have shown no increase in miscarriages, stillbirths, developmental disorders in babies born after the accident, nor have they found a link between the nuclear disaster and incidence of thyroid cancer within Fukushima Prefecture [8]. However, the results of a large-scale mental health and lifestyle survey conducted throughout the prefecture by Fukushima Medical University indicated post-traumatic psychological distress among residents of Fukushima Prefecture, both in evacuees and non-evacuees, as well as increased rates of obesity, binge drinking and alcohol use disorder, depression, and suicide. The rate of psychological distress was noted to be particularly high in children and adolescents compared to the background population [9]. These effects are more prevalent among evacuees, who face “radiation stigma” – stigma due to the fear that these individuals may contaminate others, or that they might bear children with severe birth defects due to potential exposure [10].

## Methods

### Context

In the academic literature written in English, the events of March 2011 are referred to collectively as “the Tōhoku disaster,” “the Fukushima disaster,” or “the triple disaster,” or else as variations on “3/11 [11].” However, in order to be consistent, accurate, and respectful, I will to refer to the earthquake-tsunami, the subsequent incident at the power plant, and the repercussions of all three components of the event in the same way that the research participants referred to them: the Great East Japan Earthquake (GEJE), or simply, the Earthquake. The Icahn School of Medicine at Mount Sinai and Fukushima Medical University have enjoyed a partnership since 2012 – each spring, two third-year students from Fukushima Medical University come to the Icahn School of Medicine to study endocrinology, and each summer, two students from the Icahn School of Medicine conduct research at Fukushima Medical University. A major goal of this partnership is cross-cultural exchange: students from each school have the opportunity to learn about the healthcare system, current policy issues, and the lifestyle of medical students and doctors in their host country. As I was a student at the Icahn School of Medicine at the time of the study, this existing relationship paved the way for my participant recruitment.

### Study population and recruitment

Interview participants were limited to students enrolled at Fukushima Medical University who lived in Fukushima Prefecture at the time of the Great East Japan Earthquake. Medical school provides students with a medical degree and general medicine education with which students will go on to specialize in residency. Medical education in Japan lasts 6 years and begins after high school. As a result, most students are generally somewhere between 18 to 24 years of age.

In a student body of 792 students as of May 1^st^, 2019, only 32% (253 students) came from Fukushima Prefecture [12]. Exponential non-discriminative snowball sampling, where each participant recruited could provide one or more referrals, was the main method of participant recruitment. Initially, participants were invited to participate via announcements made at the beginning of classes for year 1 to 6 students as well as through class group chats in the LINE mobile messenger app. Two reminders, spaced two weeks apart, were sent in the class group chats. The first wave of recruitment garnered 5 participants. These participants then provided recommendations for other students to attempt to recruit, garnering an additional 3 participants in the second wave of recruitment. Concurrently throughout both waves of recruitment, members of a disaster medicine interest group called “Fukushima WILL” reached out to their club members. This garnered 2 participants. Of the initial group of 253 students, 10 students agreed to engage in interviews. There were no limits on the number of participants for the study. The natural endpoint for participation coincided with the conclusion of the exchange program (August 2019). Participants were in years 1–5; no 6^th^ year students from Fukushima Prefecture were available to interview due to demanding curricular requirements during the summer months.

### Interviews

Interviews were conducted from June to August 2019. Interviews were open-ended and an interview guide was used (Appendix). The interview guide was developed by the authors, including two physicians (CK, RY), two medical students (AS, ML), two Japanese medical students at FMU, and a Japanese faculty member at FMU (SW). The interview guide was updated throughout the study as needed. The goal of the open format was to encourage the participants to reveal and discuss what *they* wanted to reveal and discuss, rather than what the researchers wanted.

Interviews took place in study rooms and classrooms on FMU’s campus. Informed consent to participate was obtained both in English and Japanese at the start of the interview. Written consent for recording and publication was obtained as well. All but one of these interviews was private – unfortunately, a private room could not be reserved for Participant 2, and there were other students in the study room at the start of the interview. Additionally, Participants 8 and 9 requested they be interviewed together, and brought three friends with them who wanted to observe. Though the researcher (AS) verified many times prior to the start of the interview that this was alright with both participants, they insisted that they would rather have their friends stay. Ultimately, the researcher (AS) favored participants’ comfort over a “sterile” interview environment.

Following consent, participants were provided with the choice of conducting the interview in English or Japanese. The choice of language depended on both the participant’s comfort with speaking English and their desire to practice. Interpretation from Japanese to English and vice versa was provided telephonically by a language access company, Pacific Interpreters. Interpreters hired by the company have passed both language proficiency and interpreter readiness tests in English (as well as Japanese in this case). Throughout the interviews, the interpreter would convert from Japanese to English or vice versa in real time. Interpreters would sometimes ask clarifying questions to either the English or Japanese speaker to more accurately translate the meaning of the speaker’s words. The researcher did not perceive a difficulty in understanding or being understood by any of the participants. During one interview involving Participants 8 and 9, an telephone interpreter through Pacific Interpreters was unavailable. Instead, a friend observing who was fluent in English served as an interpreter for Participant 8. Of the 10 participants, 5 conducted the interview in Japanese via an interpreter, and 5 conducted the interview in English.

Interview times varied from approximately 30 min to over 3 h, but the average session length was around 1.5 h. All interviews were audio recorded and were later transcribed by a researcher (AS). Only the English language portions including words spoken by the interviewer, interpreter, or participant were transcribed (i.e. not the Japanese language from the participant or interpreter). A second researcher (ML) listened to all the audio recordings along with the transcribed document to ensure no errors in transcription were made.

Throughout this study, interview participants are referred to by the order in which they interviewed – for example, the third participant to be interviewed is called “Participant 3” – in an attempt to anonymize participants. The researchers recognize the relative futility of this effort; as anthropologist Rebecca Nelson points out in her wonderfully titled essay “How Can We Hide Participants’ Identities When They’re on Pinterest?,” the ubiquity of social media and the necessarily specific identity markers of these interview participants (Fukushima Medical University students from Fukushima Prefecture) allow curious readers to “play detective,” making it difficult to respect the privacy and ethical commitments made to participants [13]. Therefore, personal and identifying information is withheld from the text whenever possible. All participants, regardless of the language spoken, are quoted in first-person – translators from Pacific Interpreters relayed the words of Japanese-speaking participants directly – with the exception of Participant 8, who was interpreted in third person rather than first.

### Methodological approach, data analysis, and rigor

The topics discussed in this text are the result of an inductive thematic analysis of the interview transcripts under the lens of ethnographic anthropology, so chosen for the discipline’s goal of exploring cultural experiences from the point of view of a study’s subject rather than from the perspective of the researcher. The researchers began the analysis while collecting the data. The inductive thematic analysis process comprised the following steps: (1) familiarizing oneself with the data, generating initial codes, (2) defining and naming interpretative codes for the entire data set into themes, (3) identifying patterns across all data to derive themes for the data set, and (4) lastly defining and naming themes. AS, ML, CK, and RY were involved in all steps of the thematic analysis and served as reviewers. This measure minimized the risk of a researcher’s individual bias, personal preconceptions, or positionality overly influencing the data analysis. Team meetings were organized frequently to share the research path and reframe objectives as needed. Credibility was obtained through prolonged engagement of all the researchers with the setting (ML and AS were in Fukushima together; CK and RY had been to Fukushima yearly for almost 10 years). Credibility was also assured by gathering rich data from the interviews, transcribing the interviews verbatim, and analyzing the data line by line.

## Findings

Three themes naturally emerged in the inductive analysis: first, that the events following the Earthquake influenced not only these students’ decisions to become doctors, but also the ways in which they hope to practice medicine in the future (Section entitled “Effect on future medical practice”). Second, that these students were motivated to share their experiences by a want to change Fukushima’s public image (Section entitled “Righting Fukushima’s public image”). And lastly, that the students viewed the opportunity to discuss their experiences through these interviews as healing, both for themselves and for the future (Section entitled “Research as healing”).

### Effect on future medical practice

All of the interview participants attributed some part of their budding medical careers – be it the medical specialties they’re interested in or their wish to study or practice in Fukushima – to their experiences following the Earthquake. For some of the students with whom I spoke, the Great Earthquake acted as a catalyst of sorts in their decisions to pursue a career in medicine. “I think,” Participant 1 observed, “what you did before you were medical student maybe makes you what you are today.” Indeed, of the 10 students interviewed, 6 cited their experiences following the GEJE as a motivating factor in applying to medical school.

Qualitative evidence supports this trend. A 2015 survey of FMU medical students who had engaged in recovery volunteer work showed that these student volunteers displayed a statistically significant increase in desire to become a physician compared to non-volunteers. Authors Anderson et al*.* acknowledge that in the years that elapsed between the Earthquake and the survey, volunteers and non-volunteers alike certainly had other experiences that influenced their professional goals [14]. However, some of the students interviewed for this study explicitly connected the Earthquake to their chosen career paths.

Some, such as Participant 1, expressed an interest in medicine prior to the Earthquake, but noted that this interest was heightened by the disaster. Participant 1 explained, “…After the earthquake, partly because my father’s job is doctor, I thought that medical is essential for daily life, I thought about it stronger than before the earthquake. That’s why I thought that I want to be a doctor.” Participant 5 similarly reported a strengthening of their pre-existing interest in medicine by the Earthquake. They explained, “I learned through this experience [of the Earthquake] that one of the most important things is helping people. That’s the biggest thing I learned through the disaster. I think it’s connected to what I want to do in the future.”

Other student interview participants viewed their experiences following the Earthquake as essential in their choice of career – Participant 8 is one such example. Though Participant 8 had worked in healthcare prior to the Earthquake as a nursing home aide, they decided to apply to medical school after coming into contact with evacuees from the exclusion zone, including the displaced residents of another nursing home in Namie, a town directly downwind of the Fukushima Daiichi Nuclear Power Plant. Participant 8 explains,, “As a fellow citizen of Fukushima Prefecture, [Participant 8] was always thinking about the…evacuation zone. The people in the evacuation zone, he want[ed] to do something for them, but he [had] a daily life, and work…so he [couldn’t] do enough for them.”

Participant 2 was the most emphatic in describing the Earthquake’s role in her choice of career. She recounted, “Before the Earthquake happened, I didn’t know what I wanted to do in the future, I had no goal or dream. But then I’d seen so many friends die, and I went through that experience, and that made me want to save lives.” She went as far as to say, “I think that if I didn’t experience the accident, there’s no way I would have gone into medicine.”

The aftermath of the Great Earthquake affected not only her decision to pursue medicine, but also her priorities as a future healthcare practitioner. After the Earthquake, she and her friends experienced several years of flashbacks and panic episodes triggered by images and videos of the disaster. “But what saved us,” she said, “was school counselors, doctors, and mental health professionals. So I [want] to be a doctor who can help, not just medically, but mental health as well. Not just physical health, but mental health as well. That’s really important.” This emphasis on an integrated approach to medicine, particularly for children and adolescents, was shared by several of the participating students. Participant 1, who is not at present planning on going into psychiatry, expressed the importance of incorporating psychiatry into his future medical practice:

*It’s important thing to have psychiatric mind, psychiatric comprehension skills. How to feel sympathy, how to open [patients’] minds, how to icebreak. I think, especially for child, like adolescent age…I think it’s kind of like 100% skill of a doctor. It’s essential to me. Psychiatry is important to every medical field…I strongly think so because I can emphasize on people’s depression and people’s negative minds. Especially in people who struggle in harder situations, like attacked by not only nature disaster but also violence. Maybe I can feel sympathy with them. I want to be a different doctor.*

Participant 1 describes most doctors as being “100% medical mind,” his shorthand for a scientific approach to the exclusion of the personal, but he describes himself as being “less medical than [his] classmates” at only 90% “medical mind,” a trait he attributes to living in Fukushima after the Earthquake. It is this trait that he hopes to use to become “a different doctor:”

*Majority of my classmates, I think…are kind of like more concentrated on their medical things, more than me. They tend to avoid to talk with ordinary people [who aren’t in medicine]…Students totally forget about the normal mind because of the exposed by the medical things. But to have sympathy with “normal” people, maybe we have to still have a kind of normal mind about, like, everything. So I don’t think 100% medical mind is a good thing.*

Participant 9 describes a similar mindset that they attribute to their experiences as a post-disaster recovery volunteer in Iwaki, the site of the Earthquake’s epicenter. Of those displaced by the Earthquake, Participant 9 states, “It’s very important to do healthcare for them. It’s also very important to chat with them, to talk with evacuees, about a lot of things – for example, their families, their hobbies, and so on – while doing treatment.” While he states the Tōhoku Earthquake did not influence his decision to pursue medicine, he says it did change his field of interest from surgery to family medicine [15]. He explained,

*When I first wanted to be a doctor, I thought “It’s cool to become a surgeon, very cool.” But after the big earthquake, my feeling changed because a lot of people were affected by a lot of hardship. If I become a surgeon, I will help one person per day. But if I become a home doctor, I will treat many people.*

All of the students with whom I spoke described a relationship between their medical field of interest and their experiences living in Fukushima following the Earthquake. While none of these students will have to decide until after they graduate from medical school, their current thoughts on specialties reveal the lasting impact of the Earthquake.

Participant 7 is currently doing research in lifestyle medicine and preventative care, which she says “also includes what to do after a nuclear incident, so I guess I’m ending up studying life after the nuclear accident, because I think it’s important to know how to prevent risks of being sick after that.” As a specialty, she’s interested in “psychiatry, because you have to know how the disaster affects the mind.” Participant 9 is interested in family medicine for the same reason as his friend Participant 8. Participant 4 is considering pediatric psychiatry, inspired by loved ones who struggled with their mental health following the Earthquake – “I want them to know that they don’t need to be ashamed,” she declared.

Participant 3 described a similarly personal motivation for his field of interest. Due to space constraints, his was the only interview conducted in public, and, though nervous about his level of proficiency, he requested we switch our conversation to English for this portion of our discussion. His friends, none of whom spoke English, were sitting at another table on the other side of the room, and he glanced at them before sharing,

*After the disaster, I saw sick people who couldn’t be treated properly, so I thought, you know, I want to provide proper care for sick people… My mother…had cancer. And the time is just when the earthquake happened. So when the earthquake happened, lots of doctors went away to other prefectures, so Fukushima Prefecture had little doctors, so...there were no people, she couldn’t get treatment… And later – she was gone. She died.*

This difficult experience was the impetus for Participant 3’s interest in a career in regenerative medicine. He explained, “Because of this disaster, we had less doctors, so patients couldn’t be treated properly. Like my mother. So the development of new technology would help patients treat themselves, by themselves, for themselves.”

Several participants also expressed a desire to stay in Fukushima to practice medicine once they graduate, out of a want to help heal their prefecture. While this is not unique to these participants – 56% of Fukushima Medical University’s last 5 graduating classes have stayed in the prefecture after graduation for their medical internships [12] – the students interviewed specifically cited a wish to help their home communities recover. Participant 2 says she wants to pursue internal medicine “since I want to go back and help the locals.” Participants 8 and 9 are both hoping to become home-visit family doctors because “Fukushima needs home doctors.” Fifth-year student Participant 6 felt that the Earthquake so transformed his plans in practicing medicine that he confided, “I think that 3/11 is good experience for my life.” He went on,

*[The Earthquake] was the trigger of my studying radiation and [now] I want to know more about Fukushima. If there were no disaster, I would want to go to some big city, but there was a disaster, so now I want to help Fukushima. It was a great experience for me. But someone who had big damage by the disaster would think that it’s an imprudent point of view.*

### Righting Fukushima’s public image

This desire to help Fukushima heal extends beyond the literal. Many individuals I spoke with in Fukushima, both in the context of interview and out, lamented the fear that many outside of the prefecture feel towards Fukushima. As Participant 2 put it, “…People, especially overseas, around the world, they think that Fukushima is a very dangerous place to live, still, and that everyone who lives in Fukushima is sick. But that’s not true. Ideas like that, they should be fixed.”

**Fig. 2:**
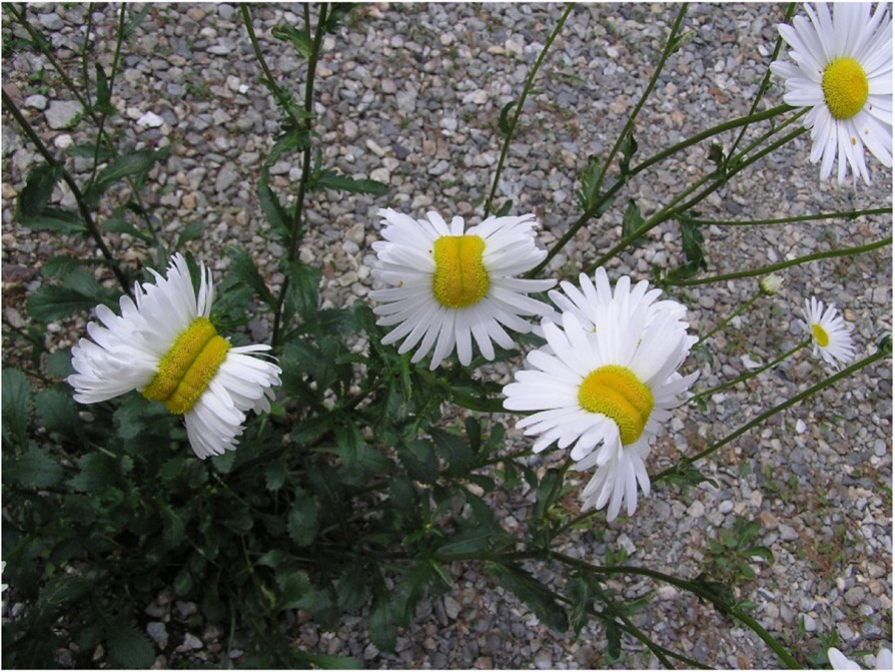
Viral photo of fascination mutation in daisies https://www.twitter.com/san_kaido/status/603513371934130176. Used with permission from Twitter user @san_kaido

I have encountered this line of thinking myself, both in the United States and in Japan. In planning my visit to Fukushima, friends – including my classmates in medical school – asked me if it was safe to be there. A bartender in Osaka confided that she would never visit Fukushima, and that her friends from Korea have even asked her if visiting Tokyo is safe, given its “proximity” to Fukushima. A few weeks into my stay, in looking for the address of a shop in downtown Fukushima called Daisy Bell, I made the mistake of running a Google search for “Fukushima daisy.” Instead of the address I had hoped for, my search returned articles with titles such as “These ‘Mutant Daisies’ Near Japan’s Nuclear Disaster Site Are Freaking Everyone Out [16],” flanked by images of a group of daisies that appear elongated, like several flowers merged together (Fig. 2). They came to the internet via Twitter user @San_kaido, who uploaded them in May 2015 with the caption “The atmospheric dose is 0.5 μSv/h at 1 m above the ground [17],” which lead many viewers to assume that the mutations displayed in the photograph were caused by nuclear radiation from the Fukushima Daiichi Nuclear Power Plant. In reality, such mutations (called “fascinations”) occur naturally worldwide, without the influence of radiation. [18].

These images, accompanied by the claim that the flowers shown had “nuclear birth defects,” were used in a 2016 study by the Stanford History Education Group to investigate the ability of Usonian high school and college students to evaluate the credibility of information that exists on the internet [19]. According to study co-director Sarah McGrew, “Most students – the vast majority – noticed none of those problems” [18]. Eighty percent of high school students accepted the picture as proof of the continued effects of radiation in Fukushima [19].

The public’s fixation on Fukushima as “the ultimate meme of nuclear power [20]” is still going strong, fed by an eager media. In April 2018, British tabloid newspaper *The Sun* ran a story with the outrageously dramatic headline “Japan ‘covering up’ Fukushima nuclear danger-zone radiation levels and blackmailing evacuees to return to radiated areas swarming with radioactive pigs and monkeys [21].” In July of that same year, Netflix aired its original docuseries *Dark Tourist*, which follows journalist David Farrier as he visits, in the words of the show’s Netflix Official Site, “unusual – and often macabre – tourism spots around the world [22].” Episode 2, “Japan,” begins by following Farrier on an organized bus tour in an unspecified location near the Fukushima Daiichi Nuclear Plant as Farrier wonders in the narration, “Is it even safe to be here?” He and his fellow “nuclear tourists” are then shown in Tomioka, anxiously watching the numbers on their Geiger counters rise, and a woman comments, “Oh my god, the level of radiation, that’s higher than around Chernobyl where no one’s allowed to go.” The group later has lunch in Namie, where Ferrier says of the food, “It may be radioactive, but it’s delicious [23].”

While it is not within the scope of this research to disprove the false rumors on the safety or lack thereof of Fukushima, it’s important to point out that the high radiation levels observed by Farrier and his fellow tourists reflect the exposure they would have received in an hour, and that the threshold for sickness against which they compared their readings is the amount of continuous exposure needed per hour to cause damage over the course of a year. In fact, Farrier likely received more radiation flying from Japan to the United States to film his next episode than he did on his tour in Fukushima [24].

The Fukushima Prefectural government and the Reconstruction Agency are upset by more than Farrier’s cavalier attitude towards the Tōhoku Earthquake (“It’s been a real buzz seeing a nuclear wasteland firsthand,” he remarks in a voice-over); they reported considering legal action due to concerns that “the video could fuel unreasonable fears related to the March 2011 disaster [25].” The episode has certainly had an impact on the nuclear tourism community: despite the government’s efforts, an estimated 100,000 foreign visited Fukushima in the year following the show’s release, many attracted by the opportunity to see what Farrier called “a nuclear wasteland [23, 26].” In reality, it’s not, and the Fukushima Prefectural government has been working hard to disabuse this notion. However, the wide-reaching impacts of the sensationalism and misinformation following the Earthquake have been so severe that some have dubbed the so-called “harmful rumors” the “fourth disaster” of the Triple Disaster [27].

The widespread view of Fukushima as dangerous, its food as radioactive, and its people as sick or even mutated is familiar to the interview participants. “Rumor itself,” Participant 8 said, “is a very evil thing, especially when there are a lot of vulnerable people in Fukushima.” Tabloid and gossip media, he argued, were equally “evil.” Participant 7 was in agreement. She observed,

*A lot of people outside Fukushima have fears about radiation, and I think those fears exaggerated and became rumors, all over the place. There was so much information after the nuclear accident. The media picked up so many stories. But I don’t think many people visit the government site [to verify information], but I don’t know. I’m sure some people went to the government site to get information, but there was just so much information on the media, and this is where many people went for their information…People inside of Fukushima, they tried to find out what really happened, but people outside of Fukushima – once you go outside of Fukushima, they’re just not interested in seriously learning the truth, so they still go by biases about Fukushima. Once you go outside of Fukushima, a lot of people have those biased ideas, but I can’t blame them because they don’t get much information.*

As an evacuee from Minamisōma, Participant 7 is passionate on the topic of misinformation on Fukushima. Since the Earthquake, she has heard these claims from peers and adults alike. She shared:

*After the incident, people were so stupid sometimes, saying things like “People from Fukushima, they have runny noses all the time,” or “They don’t live that long,” or “You shouldn’t get married to women from Fukushima.” I heard all kinds of terrible, ignorant comments. As a teenager, I was so shocked to hear that, so that’s why I’m interested in medicine now, [because] I was so shocked to hear those rumors from people. Before I went to college, I went to a prep school outside of Fukushima, and students and teachers at the prep school, those people would tell me their biased ideas, like that we’re all sick or that we all have cancer, that we all have tumors. I was so shocked to hear this. Even the teachers said that to me…You know, when I heard that, I defended Fukushima, you know, saying “It’s not like that.” I kept telling them that, but after a while, I just started ignoring those people and their words. No matter what I was going to say, they would never change their minds. Those biased ideas were already in there already, so I just stopped correcting people like that.*

However, many of the student participants felt that by sharing their experiences through these interviews with “foreign researchers,” they had the chance, as Participant 4 put it, “to tell everyone the right situation of Fukushima.” Participant 3 declared:

*Prejudice about Fukushima, I want to clear it…Any wrong information, such as the idea that Fukushima people cannot live because of the radiation – that’s not true, that’s not correct. Also, the idea that Fukushima produce is not edible, that’s not true…When the incident occurred, there was a lot of news going around which discriminated against Fukushima, and I don’t want that…There’s still a lot of prejudice about Fukushima going around, so by speaking out, I hope I can clear some of it up. This is important to me…That’s the reality, and I want people to know about it.*

Participant 6 also implored me to tell people in my “home country” about the reality of Fukushima’s situation. He sees the Earthquake as an essential time in his life and in the lives of other individuals from Fukushima, and even called the incident “our disaster” several times throughout the interview. Despite his wish for Fukushima to move forward, he feels this cannot be accomplished until the misconceptions have been cleared. He explained,

*I don’t want to keep in the past, in bad things. I want to go ahead, to forget about that disaster. But we have some impacts still from this disaster, so it is part of our, part of my life. It is part of myself, it is part of my identity. So to educate people is a good thing…I think that the information about this disaster, the information in foreign countries, it’s not exact as in Japan. So some foreign students come to Fukushima and study about it and go back to their home country and spread the exact information, it’s very important thing.*

He experienced this importance firsthand in 2018 as an exchange student in Belarus. Like its partnership with the Icahn School of Medicine at Mount Sinai, Fukushima Medical University has an “academic cooperation” with Belarusian State Medical University and Gomel Medical University, facilitated by Nagasaki University’s Atomic Bomb Disease Institute [28, 29]. Part of Belarus is still included in the Chernobyl Exclusion Zone following the Chernobyl disaster of 1986, and as Nagasaki University’s international collaborative research coordinator wrote, “There has been active attitude in Japan toward learning lessons of Chernobyl accident aftermath and countermeasures since the Fukushima Nuclear Power Plant accident [29].”

Though Participant 6’s curriculum focused on studying military medicine, shadowing thyroid surgeries, and practicing effective patient communication, what left the most lasting impression on him was how his concept of Chernobyl changed [30]. In our interview, he described,

*Before we visited there, I thought Chernobyl is very terrible, destroyed, but it is not exact information. Chernobyl is not terrible condition. And my understanding changed after I visit there, so I have good image about Belarus and about Chernobyl. [For foreign students] to come to Fukushima and go back to their home country helps them, they can have exact information. They can have a similar experience as I had in Belarus.*

He, like other participants, believes that international collaboration allows for increased opportunity to set the record straight when it comes to Fukushima.

Participant 4 had a similar experience as Participant 6, though as a host rather than as a guest. In high school, she took part in a workshop in which Fukushima students partnered with French students as they toured through the prefecture in an effort to show what Participant 4 calls “the right thing” to the visiting students. She elaborated,

*People should just know the right thing. I know the right thing because I live in Fukushima. I want to tell the world the right thing because I know the right thing…I [thought] I could tell them [the French students] the right things of Fukushima. The right things instead of harmful rumors about farmers. And I could show them the nice sightseeing places of Fukushima. And the delicious food of Fukushima.*

Participant 4 explained that she felt she had, and still has, a responsibility as a Fukushima resident to represent her prefecture and to ensure that others have an accurate understanding of the place that she calls home. She expressed a wish to replace the damaging, inaccurate ideas I may have received with positive ones, and called this wish her “duty.” She said:

*That earthquake was big turning point of my life. And my high school teacher said that in the future, we are going to be asked about 3/11 and the accident at Daiichi, so we need to know the right situation of Fukushima, and we have duty, we need to tell people around Fukushima, around Japan. We have a duty. So I realized that I have to know the situation of Fukushima, the right situation of Fukushima. Do you know the bad rumors? The bad rumors about Fukushima? ...We, the people who live in Fukushima, have to tell [others] the right things about Fukushima, I think.*

However, not all those from Fukushima Prefecture are as clear as Participant 4 as to “the right things about Fukushima.” Participant 8 explained, “[We don’t] know which was rumor and which is truth. Even the people, the local people, [we don’t] know that.” He noted that the media’s platform has been enhanced by a continued lack of trust in the government. Of his fellow Fukushima residents, he said, “They can’t trust the government’s statement…They couldn’t think that they should believe all the information from the government.” When I asked Participants 8 and 9 where they believe the mistrust of the government comes from, they discussed in Japanese for a moment and laughed before offering, “The government said that the nuclear plant was safe. But it wasn’t.”

Distrust of the government in Fukushima following the Earthquake has been well documented. The Washington Post describes “a culture of coverups and denials that contributed to the nuclear accident and continues to dog Japan’s efforts to restart its nuclear industry,” referring to the Tokyo Electric Power Company (TEPCO)’s attempt to deny that the nuclear disaster had occurred for months after the accident [27]. Subsequently, TEPCO’s president Naomi Hirose apologized for the lack of disclosure, admitting, “I would say it was a cover-up [31].” In January 2019, a member of TEPCO’s external advisory committee (and former head of the U.S. Nuclear Regulatory Commission) said at a news conference, “If TEPCO does not improve their communication, it will be very difficult for them to regain the public trust [27].”

Due to this local wariness of government information, Participant 8 expressed that unlike many of the other participants, his top priority was not to educate “not the whole world, or all Japanese people’s minds, but the people around him.” He was adamant on this point, insisting several times that he wants to enact change “in a small way” rather than on a large scale. His friend translated,

*He has a strong feeling about caring for the people in his community. He has feelings for his community, but his community is not so big. He doesn’t want to change public opinion or something. He just wants to change his people. Everyone has to care for their own.*

For some students, the desire for individuals outside of the prefecture to know “the right situation of Fukushima” stemmed from, as Participant 3 phrased it, a want “to be treated like everybody, just like anyone. Not anything special, we’re just like anyone. I want to be treated the same. Equal.” Participant 7 remembers expressing this wish as well and described being treated differently than her classmates. As a high school student, she noticed that her teacher was projecting his idea of an evacuee on her rather than recognizing her true feelings. She recounts:

*My teacher treated me like an evacuee. He felt so sorry for the kids like me in his class. As a kid, moving to my grandparent’s house, moving to the city, I was excited, but the teacher didn’t understand how I was feeling. I think the teacher pitied me. He didn’t treat me like a regular student, and when I was 14, I just wanted to be like everybody else. Teenage life by itself is confusing, but with the accident affecting that time of my life, it was all much more complicated.*

However, the motivating factor expressed most often by participants was their love of Fukushima. Participant 5’s to-the-point explanation summarizes the thoughts of many of the students. She shared,

*In general, if you see the news, you know that people are kind of horrified about the lasting effects of the nuclear accident. But, you know, I have to tell them, those effects are not as bad as they think…A lot of people [are] living here, in Fukushima, and I don’t want to present that image of Fukushima, because I love it here.*

Participant 4 was of the same mind; she believes that many residents of Fukushima such as herself want to rectify the negative image of their prefecture “because they love Fukushima,” and repeated, “I want everyone to know the situation of Fukushima.” This was the sixth time in our conversation that she’d said this – she then laughed and said of her goal, “Always the same.”

### Research as healing

For the participating students at Fukushima Medical University, these interviews appeared to serve multiple purposes. The students were clearly motivated to speak with me by a passion to change the public opinion of their prefecture, and some hoped to practice their English, or else to learn more about North American medical education. But most students also said that they wished for their interviews to be healing, both to themselves and to potential future survivors of similar disasters. Participant 7 said that because of the Earthquake, “everyone knows Fukushima,” and that the prefecture is “famous now.” “I know it’s because of the nuclear accident,” she said, “but I think we can somehow use it for good,” for both her fellow Fukushima residents, and for the future. As Participant 1 said, the interview helped to “evaporate some negative feelings,” and he declared, “I think it was a good thing for me to be interviewed.”

Participant 1 felt that this was an ideal time to discuss his experiences. He said that while there was plenty of opportunity and encouragement to talk about his thoughts and feelings in the months following the Earthquake, that has since died down, and at present, he feels such activities to be inadmissible. Of the interview, he said:

*It’s good for me to share the emotions. And, um, actually – actually three years, four years after the earthquake, the kind of atmosphere changed. Soon after the Earthquake, everybody thought, “Okay, we have to share the information and share the emotions because of the struggle with the earthquake.” But…years after the earthquake, majority people thought that we mustn’t share the emotions, we mustn’t speak about the earthquake. The main atmosphere shows that we’re already finished with the earthquake. So I think it’s kind of taboo [to] reopen old wounds. And for me, it’s only my family to share my emotions about the earthquake, about evacuation…And I think that it was a precious experience and good experience for me, to share emotions, not only with my family and relatives but also with other people.*

Participant 3 also felt that he no longer had occasions to discuss his feelings. His most memorable experience of the day of the Earthquake was leaving school in a bus and looking out the window to see “snow at the same time as thunder.” Of that sight, he said, “That was something I will never forget.” He went on, “I was with my classmates when this happened, so once and awhile, we [used to] talk about it. The five years after the incident, we’d meet up and talk about it. But not anymore.”

Participant 7 agreed that she is now in a position to benefit from an interview for different reasons than Participants 1 and 3. She explained that she decided to volunteer for an interview because “I think it’s a good time for me to look back at my past, at what happened during 3/11.” Though in our conversation she recounted difficult memories, her tone was determined, even hopeful. She explained:

*I was so afraid of doing an interview or a survey before, when I was young. I was afraid to remember my experiences, the bad things that happened. I was too young to think about what happened. I wasn’t ready to think seriously about it. But now I am old enough, and I feel I can tell you…Right now, I don’t feel anything negative [like I used to feel when] talking about what happened in my past. Actually, I have been talking to my friend who also went through the same experience – we often talk about what happened in our pasts. So right now, I feel I can talk about it more, so I think that means I’m done with it.*

Like Participants 1 and 3, she found that talking about her experiences was a crucial part of her recovery.

Participant 8 also found discussion helpful, calling the interview “a rare situation” in that it offered him a chance to reflect while moving forward. He and Participant 9 discussed in Japanese for a moment before Participant 9 relayed, “These interviews made me rethink about the disaster. Reflect. Think back. Remember my situation and know what other people have to say, and combine these things to give me new ideas and so on.” Participant 8 agreed, and his friend translated the following:

*[Participant 8] thinks that he feels like he was healed by talking about his experience…He feels very ashamed that he almost forgot about his experience with the disaster, and this is a very good opportunity to remember about the experience, what it was like…These [interviews] give us the chance to think about [the Earthquake]. Not forgetting is very important, I think. It’s not a good experience, but something good can come because of it.*

This “something” that he and his fellow interviewees hope will come of their experiences following the Earthquake is a healing in two directions, internally and externally. The internal healing appears to come from the interview itself, while the external healing will come from what they hope will be done with their words. As Participants 8 and 9’s friend translated, “They don’t want the memories of the tragedy to decay. They want people to remember the memories of tragedy…And they want to continue the memory, for the future.”

Participants 8, 9, and 1 are all members of the University’s student group Fukushima WILL, a club, Participant 1 defined via message, devoted to studying disasters and related issues as well as “radiation/radiation education in Fukushima.” Though capitalized, the name is not an acronym; Participant 1 says that the emphatic “WILL” refers to the “strong mind” of the organization, their determination to keep fresh “the situation [of the Earthquake], people’s mind, [the] difficulties.” Their hope is that by helping to spread the stories of what the residents of Fukushima went through, survivors of future disasters won’t feel as lost as they did.

This goal is not unique to members of Fukushima WILL; many participants described a hope that their words would improve conditions for survivors of future disasters. As Participant 7 said, “I hope this kind of disaster never happens again, but in case it does, I want to let everyone know, as a good example, what happened here.” Participant 8 expressed a nearly identical sentiment, saying “There are a lot of nuclear power plants around the world, so accidents will happen again in other countries, so people will need to have the right things to treat those accidents. People from Fukushima had a very interesting [unique] experience, so these things should be taken to other people.” Participant 5 also highlighted the uniqueness of the experiences of those after the Earthquake. “My life changed after the accident, so I want to make sure that people know what’s going on, what I went through,” she said. “I’d like to share my experience because…you don’t see that kind of experience often. So I’d like to share as much as possible what we went through.” She elaborated:

*…The Fukushima nuclear incident was worldwide news, it’s an experience that’s already making history, so I think it’s important for people to study what’s happened and [what is currently] happening here so they can use that experience in those difficult conditions so next time, when a similar thing happens in another country, they can use our resources. I think it’s helpful for the future. This was one of the biggest nuclear disasters in the world, in history. And I hope nothing like that happens ever again, but in case it does, I’d like to let people know what we did.*

In particular, Participant 2 hopes that by sharing his experiences, he can offer more insight into the psychological effects of such a disaster. In addition to his want to “help Fukushima get better, help people get better,” he stressed the need for increased attention to mental health in the aftermath of similar events as a reason for his participation in the interview. He explained,

*I wanted people who never had an experience like this to know about the mental health issues that me and my friends had due to the earthquake, the tsunami, and the power plant disaster. It shouldn’t be forgotten, and I don’t want people to forget…I want them to know, other people in the world, I want them to know what we experienced…I want people to understand, when there’s a nuclear accident, an atomic power accident, with a natural disaster like an earthquake, that the damage that causes to people’s mental health, that lasts many, many years. It needs to be dealt with.*

The sharing of these post-disaster stories does more than provide an opportunity for healing, or memorialize history for the future, or “lead to improved medicine,” as Participant 9 put it. The experiences of these participants are deeply personal, a part of these students’ lives. Therefore, to tell their stories is to create their legacies. In a quietly determined voice, Participant 8 – once a teacher, now one of the oldest students in his first-year class – told me and his friends why he decided to talk about what he went through:

*I may not do big things, achieve big things, in my life. But if I can tell about [my experiences] to other people, maybe they [those people] will help [other] people with hardships. These people who have had hardships, they might themselves have a high achievement, and that will give us the legacy of their achievement. So, knowing is very important, and conveying my feelings and my knowledge is very important…And people who are from other countries and other prefectures, if I give them some of the truth, maybe they will do things to help the refugees. They can do good things with that truth.*

## Discussion and conclusions

At present, Fukushima is a prefecture of dualities – at once, it is both injured and recovering; mourning and moving forward; unified with the government, and distrustful of it. The GEJE has a similarly complex significance for this study’s interview participants. For most, if not all of these students, the event was career-defining – it opened doors not only to areas of study within the field of medicine, but for some, to medicine as the field of pursuit. At the same time, the lasting, burdening effects of the Earthquake endure: trauma, loss of life and destruction of property, stigma. And yet, even these consequences act as galvanizing agents. These students stand eager, motivated, and poised to make change.

In this current study, through interviews and inductive thematic analysis, we explored the individual stories of medical students at FMU who lived in Fukushima during the time of the Great East Japan Earthquake. To our knowledge, this is one of the first studies to not only use a qualitative approach to detail common themes in the personal narratives of evacuees of the Earthquake, but also to provide a space wherein evacuees can control the narrative of the GEJE in academic literature. This study further colors our understanding of the disaster’s impact on the behavioral, societal, cultural, and political aspects of the lives of residents of Fukushima.

Our study has several strengths. The qualitative and ethnographic framework adopted for this study allows for a complex and nuanced investigation of the impact of a large-scale disaster. This approach lent itself well to broadening comparative, contextual, and cross-cultural perspectives on the GEJE. Furthermore, the focus on personal narratives and the common threads throughout these narratives adds a humanistic layer to current academic research on the topic. By placing these narratives at the forefront, participants were empowered to contribute to local and worldwide perceptions of Fukushima following the Earthquake.

There are a few limitations to our study. Firstly, our study population exclusively included medical students, a group not necessarily representative of all those affected by the Earthquake. The themes discovered in this study may not be generalizable to the entirety of the evacuee population. Secondly, as with most ethnographic studies, the richness of the data depends on both the rapport of the researcher with the subjects and the openness and honesty of the subjects. Though the researcher (AS) felt that good rapport was established through close contact with participants and their friends prior to, during, and following the interview, this is difficult to fully ascertain. Interviews were conducted in private and anonymity was stressed at the beginning of the encounters to increase the likelihood of participants being open and honest.

More chances must be given to residents of Fukushima Prefecture, both current and displaced, to provide insight into their experiences following the Great East Japan Earthquake and the associated tsunami and nuclear incident. Further ethnographic research should be conducted to expand upon the themes approached in this research, and to explore the wealth of other stories not touched here. Additionally, longitudinal studies of medical students from Fukushima Prefecture would permit more rigorous data on the impact of the Earthquake on career path. Finally, cross-cultural studies between survivors of nuclear accidents would allow for not only the healing described by the participants, but for the chance to pass on a unique expertise for potential future survivors. As the interview participants noted themselves, these stories are their legacy. 

## Data Availability

The datasets used and/or analyzed during the current study available from the corresponding author on reasonable request.

## Appendix

### Interview guide

1. What’s a typical day like for you?
2. What is your role at FMU? What do you like about FMU? Dislike?
3. Why did you decide to go to medical school/become a doctor?
4. What do you do outside of school? Where do you like to spend your free time?
5. What’s your favorite place in Fukushima?
6. How long have you lived in Fukushima?
7. What is your favorite part about living in Fukushima?
8. Do you remember the day of the Tōhoku tsunami and the Fukushima accident?
9. Can you please tell me about that day, from as early as you can remember?
10. How was your daily life affected by the accident? Your family’s daily life? Your friends’?
11. Where were you in the days/weeks/months after the accident?
12. How did the Fukushima accident affect your relationship with the medical field?
13. What medical specialties are you considering?
14. How do you feel about non-Japanese researchers conducting studies on Fukushima residents following the Fukushima accident?
15. What do you want people outside of the prefecture to know about Fukushima?
16. Is there anything else I didn’t ask that you would like to tell me?
17. Do you have any questions for me?
